# Effects of single versus dual antiplatelet therapy on the adverse events after transcatheter aortic valve implantation: A meta‐analysis

**DOI:** 10.1002/clc.23731

**Published:** 2021-10-19

**Authors:** Shengqin Yu, Shuying Zhang, Changli Yao, Jihong Liu

**Affiliations:** ^1^ Heart Center Affiliated Zhongshan Hospital of Dalian University Dalian China

**Keywords:** DAPT, meta‐analysis, SAPT, TAVI

## Abstract

Dual antiplatelet therapy (DAPT) was currently recommended for transcatheter aortic valve implantation (TAVI) postoperative management in clinical application. However, POPular‐TAVI trial showed DAPT increased the incidence of adverse events compared to single antiplatelet therapy (SAPT). Herein, we performed a meta‐analysis to investigate the effect of SAPT versus DAPT on the adverse events after TAVI. Eleven studies were available from PubMed, Embase, Cochrane Library, and Web of Science from inception to April 1, 2021. The pooled effect size was presented as relative risk (RR) with 95% confidence intervals (CIs). The sensitivity analysis was used to assess the stability of analysis results, and Begg's test was applied to evaluate the publication bias. The Cochran *Q* test and the *I* ^2^ statistic were used to evaluate the heterogeneity, and the source of heterogeneity was explored by meta‐regression. A total of 4804 patients were obtained, with 2257 in SAPT group and 2547 in DAPT group. Compared to the DAPT, SAPT was associated with the decreased risk of all‐cause bleeding (RR: 0.51, 95% CI: 0.44–0.61), major bleeding (RR: 0.53, 95% CI: 0.32–0.86), and minor bleeding (RR: 0.58, 95% CI: 0.34–0.98). There were no significant differences in mortality and myocardial infarction events, stroke events, and acute kidney injury between the two groups. SAPT was superior to DAPT in decreasing all‐cause bleeding, major bleeding, and minor bleeding, suggesting that SAPT could be preferentially recommended for TAVI postoperative management in most patients without another indication for DAPT and oral anticoagulation.

## 1 INTRODUCTION

Aortic stenosis is a common kind of valvular heart disease, affecting 2%–7% of older population.^1^, ^2^ Currently, transcatheter aortic valve implantation (TAVI) has been proved as an effective therapy to replace the conventional surgery for patients with severe aortic stenosis.^3^ However, some postoperative adverse events of TAVI cannot be ignored. Especially, thrombotic events commonly occur, with 1% being myocardial infarction (MI) and 3% being ischemic stroke, which lead to a high mortality.^4^, ^5^ Therefore, more attention should be paid to the thrombotic events after TAVI for the improvement of prognosis.

The American College of Cardiology/American Heart Association (ACC/AHA) guidelines suggest dual antiplatelet therapy (DAPT) for thrombotic events.^6^ Patients are recommended with aspirin and clopidogrel for the first 3–6 months after TAVI^6^; however, this therapy is lack of clear clinical evidence. Currently, single antiplatelet therapy (SAPT) that use aspirin alone is applied as an alternative antithrombotic treatment regimen after TAVI.^7^ Previous studies have compared the effects of SAPT and DAPT on the adverse events after TAVI, but the results remained controversial.^8^, ^9^ Hu et al. and Ahmad et al. reported that DAPT reduced the risk of thrombotic events and helped to mitigate stoke.^10^, ^11^ POPular‐TAVI trial assessed the safety between SAPT and DAPT, and results indicated DAPT was associated with a higher incidence of bleeding events.^12^ Ichibori et al. reported the similar finding that DAPT increased the risk of bleeding compared to SAPT.^7^ Rodés‐Cabau et al found that SAPT deceased the occurrence of major adverse events compared to the DAPT.^13^ Ussia et al. reported that there was no significant difference between SAPT and DAPT in death, transient ischemic attack, and bleeding events.^14^

Given that there is no consensus now, we perform a meta‐analysis to compare the effects of SAPT and DAPT on the postoperative adverse events of TAVI. Meta‐regression to explore source of heterogeneity and subgroup analysis based on study design and follow‐up time are also performed.

## 2 METHODS

### 2.1 Literature search strategy

We searched for available literatures from PubMed, Embase, Cochrane Library and Web of Science, and the deadline for searching studies was April 1, 2021. The literature retrieval was independently conducted by two researchers (S. Q. Y. and S. Y. Z.). Search strategies included: “Transcatheter Aortic Valve Implantation” OR “Transcatheter Aortic Valve Replacement” AND “single antiplatelet therapy” OR “dual antiplatelet therapy” OR “Dual Anti‐Platelet Therapy” OR “Anti‐Platelet Therapies, Dual” OR “Anti‐Platelet Therapy, Dual” OR “Dual Anti Platelet Therapy” OR “Dual Anti‐Platelet Therapies” OR “Aspirin” OR “Acetylsalicylic Acid” OR “Acid, Acetylsalicylic” OR “2‐(Acetyloxy)benzoic Acid” OR “Acylpyrin” OR “Aloxiprimum” OR “Colfarit” OR “Dispril” OR “Easprin” OR “Ecotrin” OR “Endosprin” OR “Magnecyl” OR “Micristin” OR “Polopirin” OR “Polopiryna” OR “Solprin” OR “Solupsan” OR “Zorprin” OR “Acetysal” OR “Clopidogrel” OR “SC 25989C” OR “SC 25990C” OR “SR 25989” OR “Clopidogrel‐Mepha” OR “Clopidogrel Mepha” OR “Clopidogrel Sandoz” OR “Iscover” OR “Clopidogrel Napadisilate” OR “Clopidogrel Hydrochloride” OR “PCR 4099” OR “PCR‐4099” OR “Clopidogrel Besylate” OR “Clopidogrel Besilate” OR “Clopidogrel, (+)(S)‐isomer” OR “Plavix” OR “Clopidogrel Bisulfate” OR “Hydrochloride, Prasugrel” OR “Prasugrel HCl” OR “HCl, Prasugrel” OR “CS 747” OR “747, CS” OR “CS‐747” OR “CS747” OR “Prasugrel” OR “Efient” OR “Effient” OR “LY 640315” OR “640 315, LY” OR “LY640315” OR “LY‐640315” OR “Ticagrelor” OR “Brilique” OR “AZD 6140” OR “AZD6140” OR “AZD‐6140” OR “Brilinta” OR “3‐(7‐((2‐[3,4‐difluorophenyl]cyclopropyl)amino)‐5‐(propylthio)‐3H‐(1‐3)‐triazolo(4,5‐d)pyrimidin‐3‐yl)‐5‐(2‐hydroxyethoxy) cyclopentane‐1,2‐diol”.

### 2.2 Inclusion and exclusion criteria

Studies were included based on the following criteria: (1) severe aortic stenosis patients undergoing TAVI; (2) the experimental group receiving SAPT (aspirin) and the control group receiving DAPT (aspirin plus clopidogrel); (4) randomized controlled trails (RCTs) or cohort studies; (5) studies published in English.

Studies were excluded according to the following criteria: (1) animal experiments; (2) studies without complete data; (3) conference reports, case reports, editorial materials, letters, protocols, meta‐analyses, and reviews.

### 2.3 Data extraction

Data from the eligible studies were independently extracted by two investigators (S. Q. Y. and S. Y. Z.), and a third investigator (C. L. Y.) participated to resolve disagreements. The data requested to be extracted were name of the first author, year of publication, country, study design, groups, total number of participants, age, sex, follow‐up time and outcomes.

### 2.4 Outcome variable measurement

#### 2.4.1 Primary outcomes

1. Mortality and myocardial infarction (MI) events: all‐cause death, cardiovascular death, and MI.
2. Stroke events: all stroke, disabling stroke, minor stroke, and transient ischemic attack.
3. Bleeding events: all‐cause bleeding, life‐threatening bleeding, major bleeding, and minor bleeding.

#### 2.4.2 Secondary outcomes

1. Acute kidney injury.

### 2.5 Methodological quality appraisal

Two independent investigators (S. Q. Y. and S. Y. Z.) were responsible for quality assessment. Jadad scale^15^ and revised Newcastle‐Ottawa Scale (NOS)^16^ were separately used to assess the quality of RCTs and cohort studies. The total score of Jadad scale was 7, and studies with 1–3 points were considered as low quality and 4–7 points were considered as high quality. The total score of NOS was 10, and studies were divided into low quality (<5 points) and high quality (≥5 points).

### 2.6 Statistical analysis

Stata 15.1 (Stata Corporation, College Station, TX) was applied for statistical analysis, and *p* < .05 was considered as statistical significance. The relative risk (RR) with 95% confidence intervals (CIs) was calculated to analyze the binary outcome. The Cochran *Q* test and the *I* ^2^ statistic were used to assess between‐study heterogeneity for each outcome effect size. To combine the effect amount, the fixed‐effect model was used when the heterogeneity was low (*I* ^2^ < 50%), and the random‐effect model was used when the heterogeneity was high (*I* ^2^ ≥ 50%). Based on study design and follow‐up time, subgroup analysis was used to assess the incidence of major bleeding and minor bleeding in SAPT and DAPT groups. Meta‐regression was performed to explore sources of inconsistency (*I* ^2^ ≥ 50%). Sensitivity analysis was performed for all outcomes and publication bias was assessed by Begg's test.

## 3 RESULTS

### 3.1 Study selection and baseline characteristics

**FIGURE 1:**
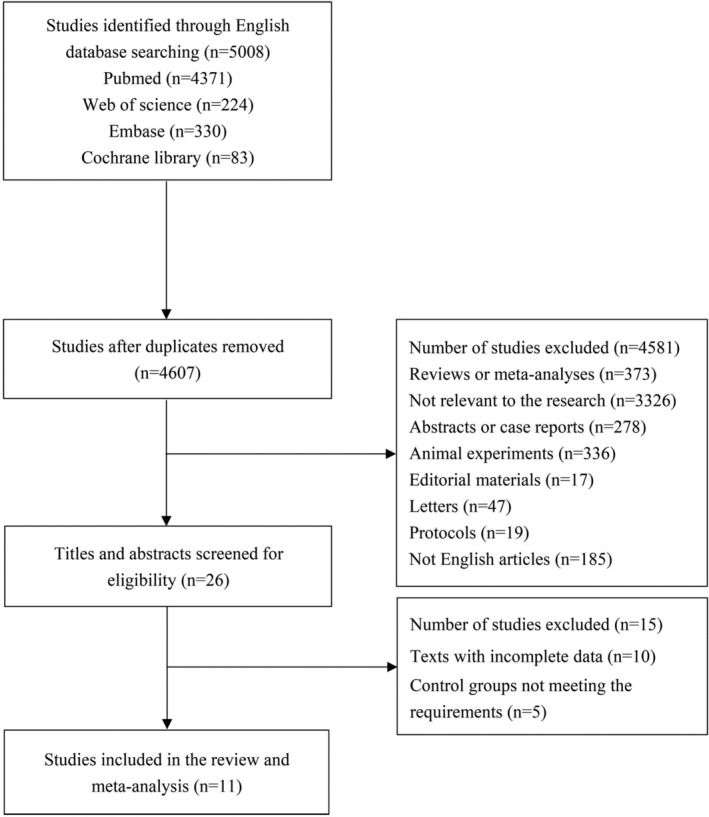
Flow chart of study selection

A total of 5008 studies were identified using the four English databases. Among which, 401 studies were eliminated as duplicates. After evaluating titles and abstracts, 4581 studies were excluded. The residual 26 texts were further assessed; of these, 15 texts were removed because of the incomplete data (*n* = 10) and control groups not meeting the requirements (*n* = 5). Finally, 11 studies (4 RCTs and 7 cohort studies)^7^, ^12^, ^14^, ^17^, ^18^, ^19^, ^20^, ^21^, ^22^, ^23^, ^24^ were included, and the flow chart of study selection was shown in Figure 1. Totally, 4804 patients were enrolled, including 2257 patients in SAPT group and 2547 patients in DAPT group. Moreover, according to the evaluation results of Jadad and revised NOS, 9 studies were of high quality and 2 studies were of low quality. Table 1 summarizes the baseline characteristics and quality assessment score of included studies.

**TABLE 1:** Baseline characteristics of included studies

Author	Year of publication	Country	Study design	Groups	Total	Age (years)	Male/female	Follow‐up (months)	Quality of literatures	Outcomes
Ussia	2011	Italy	RCT	SAPT	39	81 ± 4	16/23	6	4	a, b, c, e, f, g, i, j, k
				DAPT	40	80 ± 6	20/20			
Poliacikova	2013	UK	Cohort	SAPT	91	82	49/42	6	4	a, c, d, h
				DAPT	58	81.6	32/26			
Durand	2013	France	Cohort	SAPT	164	82.7 ± 6.3	90/74	6	5	a, c, d, e, f, g, h, i, j, k, l
				DAPT	128	84.6 ± 5.8	50/78			
Stabile	2014	Italy	RCT	SAPT	60	81.1 ± 4.8	24/36	6	6	a, b, c, e, f, j, k, l
				DAPT	60	80.2 ± 5.7	16/44			
Czerwińska‐Jelonkiewicz	2016	Poland	Cohort	SAPT	124	79.14 ± 7.39	56/68	12	6	c, i
				DAPT	352	78.92 ± 7.24	NA			
D'Ascenzo	2017	Italy	Cohort	SAPT	605	81 ± 4	256/349	12	4	a, d, h, i, j, k
				DAPT	605	81 ± 5	269/336			
Ichibori	2017	Japan	Cohort	SAPT	78	83 ± 6	28/50	12	5	i, l
				DAPT	66	84 ± 6	24/42			
Mangieri	2017	Italy	Cohort	SAPT	108	84.3 ± 7.1	46/62	12	6	a, b, c, d, i, j, k, l
				DAPT	331	82.9 ± 8.2	117/214			
Rodés‐Cabau	2017	Canada	RCT	SAPT	111	79 ± 9	59/52	3	4	a, c, d, e, f, g, i, j
				DAPT	111	79 ± 9	70/41			
Brouwer	2020	Netherlands	RCT	SAPT	331	80.4 ± 6.2	167/164	12	5	a, b, c, d, e, f, h, i, j, k
				DAPT	334	79.5 ± 6.4	174/160			
Hioki	2021	Japan	Cohort	SAPT	546	85 (81–88)	151/395	12	5	a, b
				DAPT	462	84 (81–87)	147/315			

*Note*: Data presented as mean ± SD or *n*. RCT, randomized control trail; SAPT, single antiplatelet therapy; DAPT, dual antiplatelet therapy; a, all‐cause death; b, cardiovascular death; c, myocardial infarction; d, all stroke; e, disabling stroke; f, minor stroke; g, transient ischemic attack; h, all‐cause bleeding; i, life‐threatening bleeding; j, major bleeding; k, minor bleeding; l, acute kidney injury.

### 3.2 Mortality and MI events

**FIGURE 2:**
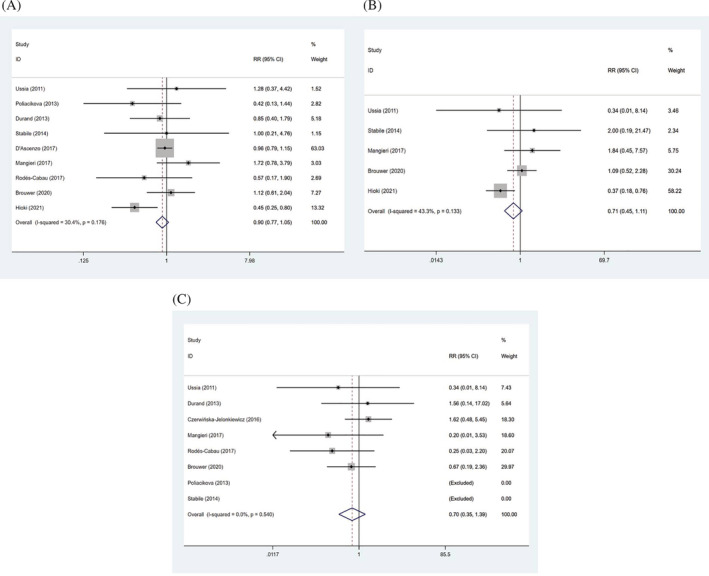
Forrest plots of all‐cause death (A), cardiovascular death (B), and myocardial infarction (C)

Table 2 shows no significant difference in all‐cause death between the two groups (RR: 0.90, 95% CI: 0.77–1.05, *p* = .183) (Figure 2A). The cardiovascular death of the two groups was not statistically significant (RR: 0.71, 95% CI: 0.45 to 1.11, *p* = .132) (Figure 2B). Also, the incidence of MI in SAPT group showed no statistical difference from DAPT group (RR: 0.70, 95% CI: 0.35–1.39, *p* = .306) (Figure 2C).

**TABLE 2:** Meta‐analysis results of outcomes between SAPT and DAPT

Outcomes	RR (95% CI)	*p*	*I* ^2^
Mortality and MI events			
All‐cause death	0.90 (0.77, 1.05)	.183	30.4
Cardiovascular death	0.71 (0.45, 1.11)	.132	43.3
Myocardial infarction	0.70 (0.35, 1.39)	.306	0.0
Stroke events			
All stroke	0.69 (0.45, 1.08)	.102	0.0
Disabling stroke	0.88 (0.39, 1.99)	.763	0.0
Minor stroke	0.73 (0.37, 1.43)	.354	0.0
Transient ischemic attack	0.90 (0.13, 6.23)	.911	0.0
Bleeding events			
All‐cause bleeding	0.51 (0.44, 0.61)	<.001	47.5
Life‐threatening bleeding	0.55 (0.28, 1.08)	.083	73.8
Major bleeding	0.53 (0.32, 0.86)	.011	58.7
Minor bleeding	0.58 (0.34, 0.98)	.044	63.3
Acute kidney injury	0.83 (0.32, 2.15)	.699	65.1

Abbreviations: CI, confidence interval; DAPT, dual antiplatelet therapy; MI, myocardial infarction; RR, relative risk; SAPT, single antiplatelet therapy.

### 3.3 Stroke events

**FIGURE 3:**
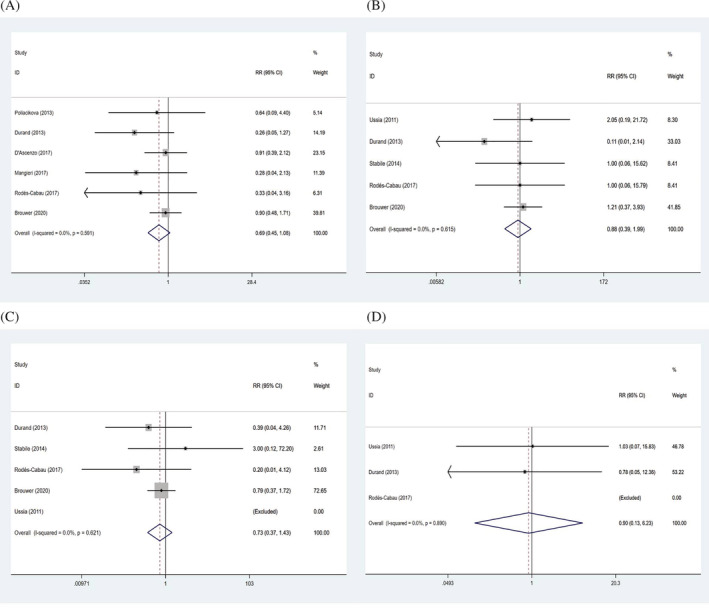
Forrest plots of all stroke (A), disabling stroke (B), minor stroke (C), and transient ischemic attack (D)

For stroke events, results were shown in Table 2, indicating that no statistical significance was found between the two groups in the incidence of all stroke (RR: 0.69, 95% CI: 0.45–1.08, *p* = .102) (Figure 3A), disabling stroke (RR: 0.88, 95% CI: 0.39–1.99, *p* = .763) (Figure 3B), minor stroke (RR: 0.73, 95% CI: 0.37–1.43, *p* = .354) (Figure 3C), and transient ischemic attack (RR: 0.90, 95% CI: 0.13–6.23, *p* = .911) (Figure 3D).

### 3.4 Bleeding events

**FIGURE 4:**
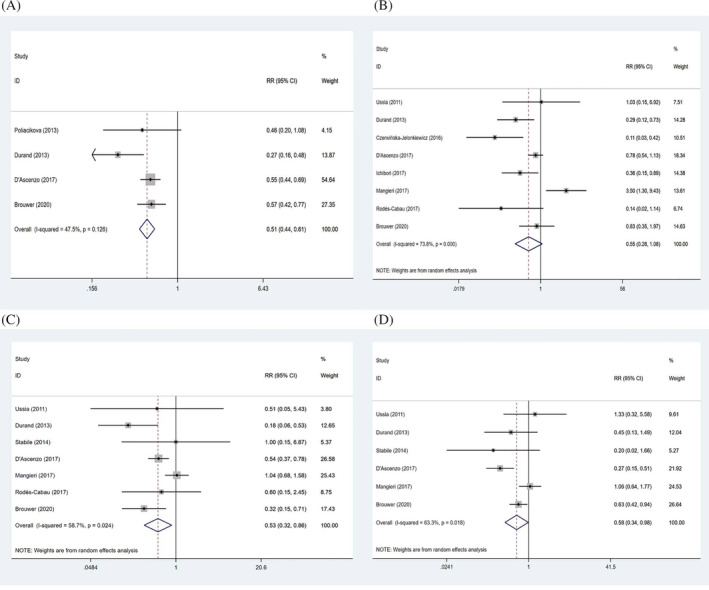
Forrest plots of all‐cause bleeding (A), life‐threatening bleeding (B), major bleeding (C), and minor bleeding (D)

Table 2 displays the analysis results of bleeding events between the two groups. Compared to DAPT, SAPT group showed a 49% reduction in all‐cause bleeding (RR: 0.51, 95% CI: 0.44–0.61, *p* < .001) (Figure 4A), while it was not significantly correlated with the decreased risk of life‐threatening bleeding (RR:0.55, 95% CI: 0.28–1.08, *p* = .083) (Figure 4B). Moreover, patients accepting SAPT had a lower incidence of major bleeding (RR: 0.53, 95% CI: 0.32–0.86, *p* = .011) (Figure 4C). Similarly, SAPT decreased the risk of minor bleeding compared with DAPT (RR: 0.58, 95% CI: 0.34–0.98, *p* = .044) (Figure 4D).

### 3.5 Acute kidney injury

**FIGURE 5:**
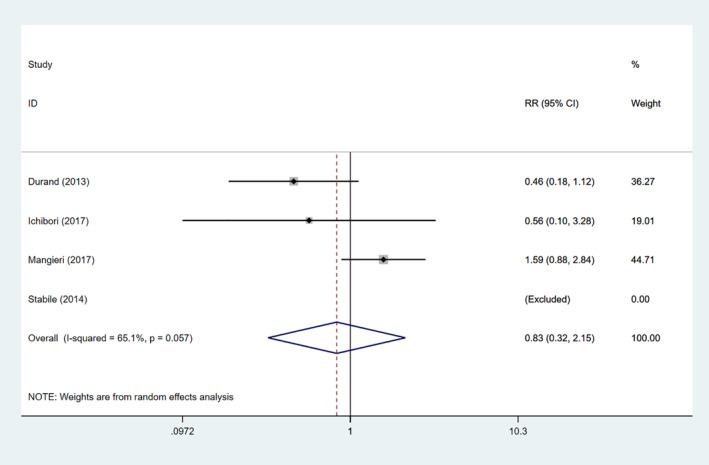
Forrest plot of acute kidney injury

The results of meta‐analysis were summarized in Table 2. Four studies were included to compare the effect of SAPT and DAPT on acute kidney injury, and random‐effect model was used. The pooling data suggested that no remarkable significance was observed between the two groups in the occurrence of acute kidney injury (RR: 0.83, 95% CI: 0.32–2.15, *p* = .699) (Figure 5).

### 3.6 Meta‐regression and subgroup analysis

**FIGURE 6:**
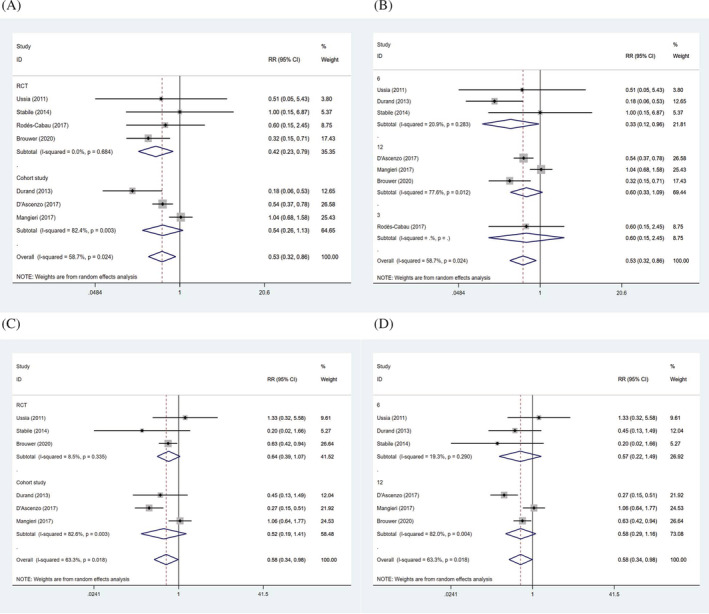
Forrest plots of study design (A) and follow‐up time (B) for major bleeding, and study design (C) and follow‐up time (D) for minor bleeding

To explore the source of heterogeneity among studies for life‐threatening bleeding, major bleeding and minor bleeding, meta‐regression analysis was performed based on study design and follow‐up time. The results showed that heterogeneity among the studies was not associated with study design and follow‐up time (Table 3). Results of SAPT versus DAPT on adverse outcomes in different subgroups were shown in Table 4. SAPT decreased the risk of major bleeding compared to DAPT in RCT articles (RR: 0.42, 95% CI: 0.23–0.79, *p* = .007), while cohort studies presented no differences between the two groups (RR: 0.54, 95% CI: 0.26–1.13, *p* = .100) (Figure 6A). Our findings also showed that the incidence of major bleeding was lower in SAPT group at 6 months follow‐up (RR: 0.33, 95% CI: 0.12–0.96, *p* = .041), but no significance at 3 months (RR: 0.60, 95% CI: 0.15–2.45, *p* = .477) and 12 months (RR: 0.60, 95% CI: 0.33–1.09, *p* = .096) (Figure 6B). Either cohort studies (RR: 0.52, 95% CI: 0.19–1.41, *p* = .198) or RCTs (RR: 0.64, 95% CI: 0.39–1.07, *p* = .087) did not show the significance between the two groups regarding to minor bleeding (Figure 6C). Similarly, the difference was not found at follow‐up of 6 months (RR: 0.57, 95% CI: 0.22–1.49, *p* = .250) or 12 months (RR: 0.58, 95% CI: 0.29–1.16, *p* = .123) (Figure 6D).

**TABLE 3:** The results of meta‐regression analysis based on study design and follow‐up time

Variables	Coef	SE	t	*p*	95% CI	
Life‐threatening bleeding						
Study design (Cohort study vs. RCT)	−0.004	1.237	−0.00	.998	−3.438	3.430
Follow‐up (6 months vs. 3 months)	0.458	2.095	0.22	.837	−5.357	6.274
Follow‐up (12 months vs. 3 months)	0.984	2.032	0.48	.653	−4.656	6.625
Constant	0.143	1.667	0.09	.936	−4.487	4.772
Major bleeding						
Study design (Cohort study vs. RCT)	0.213	0.469	0.45	.681	−1.280	1.706
Follow‐up (6 months vs. 3 months)	−0.320	0.949	−0.34	.758	−3.341	2.701
Follow‐up (12 months vs. 3 months)	−0.083	0.879	−0.09	.931	−2.880	2.714
Constant	0.600	0.765	0.78	.490	−1.834	3.034
Minor bleeding						
Study design (Cohort study vs. RCT)	−0.062	0.472	−0.13	.904	−1.563	1.440
Follow‐up (12 months vs. 6 months)	−0.034	0.559	−0.06	.955	−1.812	1.744
Constant	0.745	0.534	1.39	.258	−0.956	2.446

Abbreviations: CI, confidence interval; RCT, randomized control trail.

**TABLE 4:** The effects of DAPT versus SAPT on the adverse events based on different study designs and follow‐up time

Outcomes	RR (95% CI)	*p*	*I* ^2^
*Major bleeding*			
Study design			
RCT	0.42 (0.23, 0.79)	.007	0.0
Cohort study	0.54 (0.26, 1.13)	.100	82.4
Follow‐up			
3 months	0.60 (0.15, 2.45)	.477	NA
6 months	0.33 (0.12, 0.96)	.041	20.9
12 months	0.60 (0.33, 1.09)	.096	77.6
*Minor bleeding*			
Study design			
RCT	0.64 (0.39, 1.07)	.087	8.5
Cohort study	0.52 (0.19, 1.41)	.198	82.6
Follow‐up			
6 months	0.57 (0.22, 1.49)	.250	19.3
12 months	0.58 (0.29, 1.16)	.123	82.0

Abbreviations: CI, confidence interval; DAPT, dual antiplatelet therapy; NA, not available; RCT, randomized control trail; RR, relative risk; SAPT, single antiplatelet therapy.

### 3.7 Sensitivity analysis and publication bias

Sensitivity analysis was implemented via sequentially removed single study and reanalyzing the remaining dataset to test the strength of results. The stability and reliability of this meta‐analysis were confirmed by the similar heterogeneity before and after the study removal (Table 2). In addition, the result of Begg's test showed no publication bias in the analysis of all‐cause death (*Z* = −0.10, *p* = 1.000).

## 4 DISCUSSION

Our meta‐analysis included 11 studies comparing the effects of SAPT and DAPT on the adverse events in severe aortic stenosis patients who underwent TAVI. Overall results presented that SAPT was superior to DAPT in decreasing all‐cause bleeding, major bleeding, and minor bleeding. Either SAPT or DAPT did not show better efficacy in all‐cause death, cardiovascular death, MI, all stroke, disabling stroke, minor stroke, transient ischemic attack, life‐threatening bleeding, and acute kidney injury. Considering the higher bleeding risk and drug abuse problem of DAPT, our results suggested SAPT as the appropriate antiplatelet therapy for most patients who did not have the indication for DAPT or oral anticoagulation after TAVI.

The successful clinical introduction of TAVI is of great importance in the treatment of severe aortic stenosis.^25^ Because of the frequent transcatheter heart valve thrombosis, concern for antithrombotic therapy after TAVI has been increasingly important. Current clinical practice of post‐TAVI antithrombotic therapy is still based on experience and/or authority. Despite the lack of evidence, ACC/AHA guidelines recommend DAPT that used clopidogrel in addition to aspirin for 3–6 months after TAVI according to the clinical experience of coronary stents.^6^ Aspirin, an antiplatelet drug, has been used to inhibit platelet aggregation and prevent the formation of thrombosis after transient ischemic attack, MI, artificial heart valve or other operations.^26^ Clopidogrel is also an antiplatelet drug and used to prevent and treat heart, brain and other arterial circulation disorders caused by high platelet aggregation, such as stroke, MI and confirmed peripheral artery disease.^27^ Prior to the completion of valve endothelialization, a temporary enhanced antiplatelet regimen over one drug is considered to reduce the risk of stent‐mediated thromboembolism. Sharma et al. reported that postoperative thromboembolic events and risk of bleeding were still the significant challenge for patients undergoing TAVI.^28^ After TAVI, up to 15% of patients occurred major bleeding at 1 year, and DAPT was found to result in an increased bleeding risk compared to SAPT.^29^, ^30^ Also, the ARTE trial presented a lower incidence of bleeding correlated with SAPT than with DAPT.^13^ Similarly, our meta‐analysis showed SAPT decreased the risk of all‐cause bleeding, major bleeding and minor bleeding compared to DAPT.

Some observational studies have indicated that DAPT lacks any beneficial effects to prevent cardiovascular and cerebrovascular events after TAVI compared to SAPT.^19^, ^21^, ^23^ Two RCTs have also showed the similar results. Ussia et al. found that no differences in the major ischemic stroke between the two groups.^14^ Stabile et al. showed that the incidence of major stroke was similar in the two groups.^22^ Our results showed no differences between the groups in the stroke events, including all stroke, disabling stroke, minor stroke, and transient ischemic attack. A retrospective review from a dedicated TAVI database of a single high‐volume center in Milan reported that no significant difference was found in all‐cause mortality and cardiovascular mortality between DAPT and SAPT.^19^ Moreover, the incidence of thromboembolic events of MI in the two groups showed no significant difference.^7^ Accordingly, our results found that the incidence of all‐cause death, cardiovascular death and MI was not significantly different in the two groups. Moreover, the significance in life‐threating bleeding between the two groups was not found in our meta‐analysis, which was in accordance with the studies from D'Ascenzo et al. and Ullah et al.^20^, ^31^ Also, our study found the similar result as the reports of Durand et al. and Stabile et al. that DAPT was not superior to SAPT in acute kidney injury.^22 23^ Although SAPT was not superior to DAPT in decreasing the risk of mortality and MI events, stroke events, life‐threatening bleeding, and acute kidney injury, the use of SAPT avoided drug abuse and mitigated the economic burden of patients and their families. In addition, clopidogrel caused more damage to patients' body with a higher risk of diarrhea and rash than aspirin,^32^ supporting that DAPT with clopidogrel plus aspirin brought more toxicity than SAPT with aspirin alone.

Our meta‐analysis showed a clear benefit of SAPT for TAVI postoperative management. In addition, meta‐regression based on study design and follow‐up time was performed to explore the heterogeneity. However, some limitations were existed. First, our study combined RCTs and cohort studies, and the heterogeneity might exist among the included studies. Second, our study was lack of adjustment for confounders in baseline characteristics and comorbidities of the included patients across groups.

In conclusion, our meta‐analysis indicated that SAPT decreased the incidence of all‐cause bleeding, major bleeding and minor bleeding. Although SAPT was not superior to DAPT in life‐threatening bleeding, mortality and MI events, stroke events, and acute kidney injury, it avoided the drug abuse, and mitigated the damage to patients' bodies and the economic burden to their family members. Based on available data, SAPT was preferred after TAVI for most patients who were absent another indication for DAPT or oral anticoagulation.

## CONFLICT OF INTEREST

The authors declare no conflict of interest.

## Data Availability

The datasets used and/or analyzed during the current study are available from the corresponding author on reasonable request.
